# Different Doses of Calcium Supplementation to Prevent Gestational Hypertension and Pre-Eclampsia: A Systematic Review and Network Meta-Analysis

**DOI:** 10.3389/fnut.2021.795667

**Published:** 2022-01-17

**Authors:** Dexin Chen, Hong Wang, Xing Xin, Long Zhang, Aihong Yu, Shuwen Li, Rongxia He

**Affiliations:** Department of Obstetrics, The Second Hospital of Lanzhou University, Lanzhou, China

**Keywords:** calcium supplementation, gestational hypertension, preeclampsia, randomized controlled trials, network meta-analysis, systematic review

## Abstract

**Objective:**

Calcium supplementation can prevent gestational hypertension and pre-eclampsia. However, besides the non-consensus of existing studies, there is a lack of evidence regarding the optimal dosing of calcium.

**Method:**

Eight electronic databases, namely, the Cochrane Library, PUBMED, Web of Science, EMBASE, WANGFANG, VIP, CBM, and CNKI, were searched. The studies were retrieved from inception to July 13, 2021. Two researchers independently screened the literature, extracted data, and evaluated the methodological quality based on the inclusion criteria. In particular, the calcium supplementation doses were divided into three groups, namely, the high-dose (≥1.5 g), medium-dose (1.0–1.49 g), and the low-dose group (<1.0 g). The participants were also divided into high-risk and low-risk groups, according to the risk of developing gestational hypertension and pre-eclampsia.

**Results and Discussion:**

A total of 48 studies were incorporated into the final analyses. All doses of calcium supplementation reduced the incidence of gestational hypertension in the low-risk population (low dose - three studies; medium dose- 11 studies; high dose- 28 studies), whereas the medium-dose (three studies) reduced the incidence of gestational hypertension in high-risk groups. Moreover, a medium dose of calcium supplementation had the maximum effect in reducing gestational hypertension in low-risk and high-risk populations. The medium (three studies) and high doses (13 studies) of calcium supplementation reduced the incidence of pre-eclampsia in the low-risk groups. However, a medium-dose calcium supplementation maximally prevented pre-eclampsia in the low-risk population. The authenticity and reliability of the results were reduced due to the limitations of contemporary studies in terms of experimental design, result measurement, statistics, and evidence quality. Therefore, high-quality studies with larger sample size are required to evaluate further the effect of calcium supplementation in preventing gestational hypertension and pre-eclampsia.

## Introduction

Hypertensive disorders of pregnancy (HDP) are one of the three leading causes of global maternal morbidity and mortality. The incidence of HDP increased from 16.30 to 18.08 million globally, with a total increase of 10.92% from 1990 to 2019 (1). HDP includes gestational hypertension, pre-eclampsia/eclampsia, chronic hypertension, and chronic hypertension complicated with pre-eclampsia/eclampsia (2). Gestational hypertension and pre-eclampsia are the principal causes of increased mortality as well as maternal, neonatal, and fetal morbidity (1). Globally, 10–15% of maternal deaths and 16% of stillbirths are associated with gestational hypertension, whereas 5% are related to pre-eclampsia (3, 4). In addition, pregnant women with gestational hypertension and pre-eclampsia have a significantly increased risk of serious complications such as placental abruption, organ failure, and disseminated intravascular coagulation (5, 6).

Moreover, as the condition progresses, pre-eclampsia can quickly develop into eclampsia, which leads to life-threatening convulsions and coma. Notably, gestational hypertension and pre-eclampsia are known to threaten the maternal and newborn's health significantly, which eventually causes considerable suffering and substantial economic burden on patients (7). Meanwhile, in the latest international guidelines, the drugs are recommended for the management of HDP. However, they carry considerable risk in pregnant women and fetuses (8, 9). Therefore, early implementation of preventive measures is essential to decrease the prevalence of gestational hypertension and pre-eclampsia.

Numerous epidemiological investigations have revealed that insufficient calcium intake leads to a significant increase in morbidity and mortality during gestational hypertension and pre-eclampsia (10, 11). In addition, some studies have shown that large amounts of parathyroid hormone and renin induce vasoconstriction, which could lead to an increase in blood pressure in the absence of calcium (12). Also, some studies indicated that calcium supplementation increases the nitric oxide synthesis to reduce the contractile ability of a vascular smooth muscle, thereby lowering blood pressure (12, 13). Multiple randomized controlled clinical trials (RCTs) evaluated the efficacy of calcium supplementation for gestational hypertension and pre-eclampsia (14–16). However, their results were inconsistent. For example, the results of Hofmeyr et al. showed that high-dose calcium supplementation (≥1 g/day) might reduce the risk of pre-eclampsia and preterm birth, particularly in women with low calcium diets (low-quality evidence) (10). However, Levine et al. revealed that the daily supplementation of 2,000 mg calcium had no significant effect on the incidence of gestational hypertension and pre-eclampsia. The study inferred that the possible reason was that the people in the study had a high dietary calcium intake (>1,100 mg/day) (17). In contrast, multiple meta-analyses showed that calcium supplementation could prevent gestational hypertension and pre-eclampsia. In addition, a study performed on a low dietary calcium–based population (risk of developing HDP was low) (18) suggested that all pregnant women should take calcium supplementation to prevent HDP. Conversely, Patrelli et al. argued that calcium supplementation should only aim at high-risk groups and not at all pregnant women (19). Overall, there still exists non-consensus in calcium supplementation for HDP. Therefore, it was necessary to conduct a systematic review to explore the efficacy of calcium supplementation for preventing gestational hypertension and pre-eclampsia.

Results of multiple meta-analyses showed that (10, 18, 20) calcium supplementation during pregnancy could effectively reduce the incidence of gestational hypertension and pre-eclampsia. However, the current studies have unified the population with the different doses as the experimental group compared with the placebo group. Moreover, combining the results of various doses results in considerable heterogeneity, which affects the reliability of meta-analysis results. Therefore, we divided different doses of calcium supplementation into high, medium, and low groups, respectively. Then, the calcium supplementation groups were compared with the placebo group to explore the effective dose for preventing gestational hypertension and pre-eclampsia. Meanwhile, due to the lack of direct comparison between different doses of calcium supplementation, our study indirectly compared the effectiveness of different doses in reducing gestational hypertension and pre-eclampsia through network meta-analysis to provide evidence of the best dose for current disease prevention.

## Materials and Methods

### Inclusion and Exclusion Criteria

The following inclusion and exclusion criteria were used based on the definition of participants, interventions, comparisons, outcomes, and study (PICOS) in the Cochrane Systematic Review Manual.

#### Participants

Pregnant women without gestational hypertension and pre-eclampsia were included, with no limitation to whether people were at a high risk of HDP. Based on the risk factors of HDP, pregnant women were divided into two subgroups: High-risk group: Pregnant women with calcium deficiency, a history of gestational hypertension or pre-eclampsia, a positive roll-over test, a positive angiotensin-sensitivity test, or a high-risk factor defined in the original study (10). Low-risk group: Our study defines healthy pregnant women as a low-risk population. Now onward, the “high-risk” or “low-risk” groups addressed anywhere in our study are the participants with high or low risk factors for diseases.

#### Interventions

The test group was treated with calcium supplementation. The dose and the dose setting were described in the published literature (21). Furthermore, the test groups were divided into high- (≥1.5 g), middle- (1.0–1.49 g), and low-dose groups (<1.0 g).

#### Comparisons

The control group was treated with a placebo or blank control.

#### Outcomes

The incidence of gestational hypertension and pre-eclampsia.

#### Types of Studies

Randomized controlled trials.

#### Exclusion Criteria

Studies of calcium combined with other supplements were excluded (in the case of the calcium and other supplements, where calcium and placebo groups were included, we only extracted the results of the calcium and placebo groups); repeatedly published studies; non-Chinese and English literature; and no access of full text and/or studies with incomplete data.

### Retrieval Strategy

We searched scientific databases such as the PubMed, Ovid-Embase, The Cochrane Library, Web of Science, China National Knowledge Infrastructure (CNKI), Chinese Scientific Journal Database (CSJD-VIP), Wanfang Database, and the China Biomedical Literature Database (CBM). The relevant literature was retrieved from inception to July 13, 2021. The search terms were (pregnancy-induced hypertension OR pregnancy-induced hypertension OR gestational hypertension OR chronic hypertension with superimposed pre-eclampsia OR eclampsia OR pre-eclampsia OR hypertensive disorder complicating pregnancy OR pregnancy hypertension OR HDCP OR hypertension of pregnancy OR pregnancy with chronic hypertension) AND (calcium OR Ca). Appendix 1 describes the detailed search strategies of each database.

### Literature Screening and Data Extraction

Two trained researchers (Xing Xin, Long Zhang) selected the articles and stringently extracted the data based on the inclusion/exclusion criteria, and the selections were cross-checked. In the case of disagreement, a third researcher (Rongxia He) settled the conflict with a common consensus. Data were extracted according to the pre-established full-text data extraction checklist, including (1) Basic characteristics of studies such as authors, publication years, country, source and age of patients, start time of calcium supplementation, disease risk, dietary calcium intakes, and the number lost for follow-up. (2) Key elements of bias risk assessment. (3) Outcome measures: The incidence of gestational hypertension and pre-eclampsia.

### The Risk of Bias Among Included Studies

Using the RCT bias risk assessment tool recommended by the Cochrane Handbook for Systematic Reviews of Interventions 5.1.0 (22), two trained researchers (Aihong Yu, Shuwen Li) independently evaluated and cross-checked the inherent risk of bias in the included studies. The examples of inherent bias are as follows: selection bias, performance bias, attrition bias, follow-up bias, reporting bias, and other biases. Differences in opinion were negotiated or settled by a third researcher (Rongxia He). Answers to assessment questions (tools) were either “yes” to indicate a low risk of bias or “no” to indicate a high risk of bias. Whereas, answer of “unclear” was assigned to items for which a “yes” or “no” answer was not clear.

### Statistical Analysis

STATA 16 software was used for statistical analysis. Risk ratio (RR) was used to study the effect analysis statistic for dichotomous variables and the 95% CI as the effect amount. Heterogeneity of results between studies was assessed by a Chi-square test (The significant level for heterogeneity test was *P* = 0.1.). Also, *I*^2^ was used to judge the degree of heterogeneity. The fixed-effect model was used for meta-analysis if the research results were not statistically different. Conversely, if there were statistical heterogeneity, the source of heterogeneity was further analyzed, and the random-effects model was used for meta-analysis after the exclusion of evident clinical heterogeneity. The significance level (*p*-value) for tests was set at 0.05.

GeMTC-0.14.3 software based on the Bayesian model was used for statistical analysis. The software used Markov chain–Monte Carlo (MCMC) to prioritize and evaluate the data to achieve reticular meta-analysis. The conditions set by the network meta-analysis were as follows: Number of chains: 4, Tuning iterations: 20,000, Simulation iterations: 50,000, Thinning interval: 10. Inference samples: 10,000, Variance scaling factor: 2.5. The deviation information criterion value of the random effect model and fixed effect model were compared to analyze the fitting degree of the model. The risk ratio (RR) was selected as statistics for effect, and a 95% CI was used. The network meta-analysis used the concordance model, which was statistically significant. The node analysis model was used for the inconsistency test; if *P* > 0.05, there was no evidence to prove the direct and indirect comparison inconsistencies. The convergence of network meta-analysis was tested by the potential scale reduction factor (PSRF). If PSRF was close to 1, the convergence of this study was good, and the conclusion of the meta-analysis was reliable. Also, the network group commands were used for data pre-processing based on STATA 16 software to compare the outcome indicators of the network relationship between the intervention measures.

## Results

### Systematic Search Outcomes

**Figure 1:**
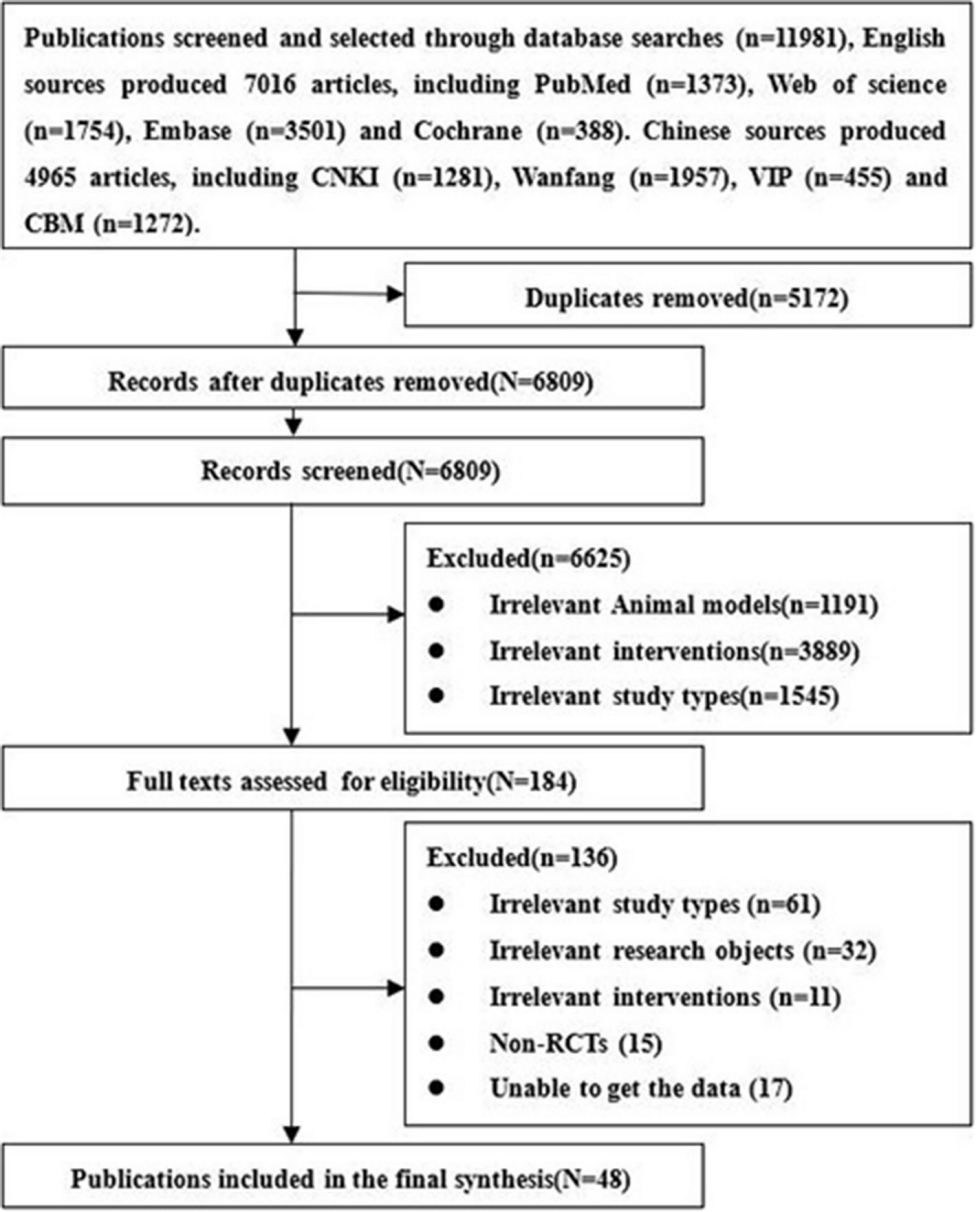
Flow chart of literature screening.

A total of 11,981 related articles including 4,965 Chinese and 7,016 English records were obtained. After excluding the literature based on the exclusion criteria, eventually 48 studies were included. The studied 48 studies include 24 Chinese articles (23–46) and 24 English articles (14–17, 47–66). The entire screening process is summarized in Figure 1.

### Basic Information for Inclusion in the Study

A total of 48 randomized controlled trials were included for analysis. The number of patients ranged from 30 (54) to 9,178 (55); Age ranged between the 16.1 (58) and 39 years (29); Of these, pregnant women in the eight studies (14, 25, 33, 52, 54, 57, 65, 66) had high-risk factors for HDP, as they had risk factors such as BMI ≥ 24 (25), calcium deficiency (33), a history of preeclampsia (65) and a positive roll-over test (52, 54, 57); Calcium supplementation initiation range was (59) 12–28 weeks (54, 57); Only nine studies (14, 25, 40, 49, 50, 52, 53, 58, 59) reported the dietary calcium intake, ranging from 85.71 (59) to 1,500 mg (14); Calcium supplementation in the test group ranged from 0.12 (63) to 2.0 g (64). The basic information of the included studies are shown in Appendix 2.

### Risk of Bias Assessment Results

**Figure 2:**
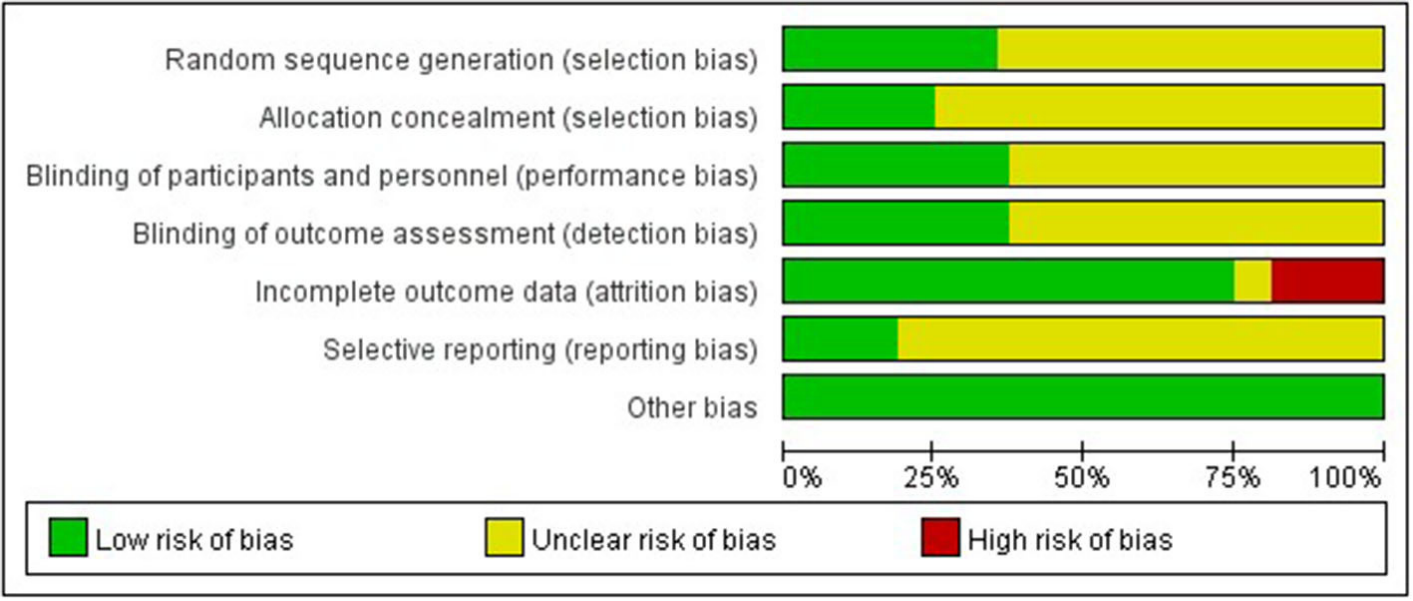
Risk of bias assessment results.

Among the included 48 studies, only 17 studies (14, 16, 17, 48–51, 53, 54, 56–58, 60, 62, 64–66) reported the specific random grouping. In addition, only 12 studies (14, 48–50, 53, 54, 56–58, 62, 65, 66) described the detailed methodology for hiding the random distribution sequences, leading to high risk of selection bias. Only 18 studies (14–17, 48–50, 52–54, 56–58, 61, 62, 64–66) implemented the blinding methods for research objects and researchers for preventing the exposure of intervention measures. Besides, only 18 studies (14, 17, 48–59, 62, 64–66) implemented the blinding methods for outcome evaluators to ensure the objectivity of the obtained experimental results. Therefore, implementation and measurement bias was at high risk. Furthermore, a risk of loss of follow-up bias was seen as there was a loss of follow-up in nine studies (14, 15, 17, 49, 52, 56, 60, 62, 64). Only nine studies (14–16, 48, 50, 62, 64–66) reported research protocols, leading to a high-risk of reporting bias (Figure 2).

### Meta-Analysis Results

#### Gestational Hypertension (Low Risk)

A total of 37 studies (16, 17, 23, 24, 26–32, 34–46, 48–50, 52, 53, 55–57, 61–65) reported the effect of calcium supplementation on preventing the gestational hypertension.

The traditional meta-analysis showed that the different doses of calcium supplementation reduced the gestational hypertension incidence [low dose: *n* = 3 studies, RR = 0.27 (0.16, 0.46); medium dose: *n* = 11 studies, RR = 0.35 (0.26, 0.49); high dose: *n* = 28 studies, RR = 0.48 (0.40, 0.58)] (Table 1).

**Table 1:** Results of a traditional meta-analysis of gestational hypertension.

**Gestational hypertension**	**Risk level of disease**	**Number of studies**	**Model**	**RR**	***I*-squared**	***P***
Low dose vs. Placebo	Low	3	Fixed	0.27 [0.16, 0.46]	0.0%	0.397
Medium dose vs. Placebo	Low	11	Fixed	0.35 [0.26, 0.49]	0.0%	0.663
High dose vs. Placebo	Low	28	Random	0.48 [0.40, 0.58]	79.6%	≤ 0.01
Medium dose vs. Placebo	High	3	Fixed	0.16 [0.06, 0.44]	0.0%	0.584
High dose vs. Placebo	High	2	Fixed	0.34 [0.14, 0.84]	57%	0.127

**Figure 3:**
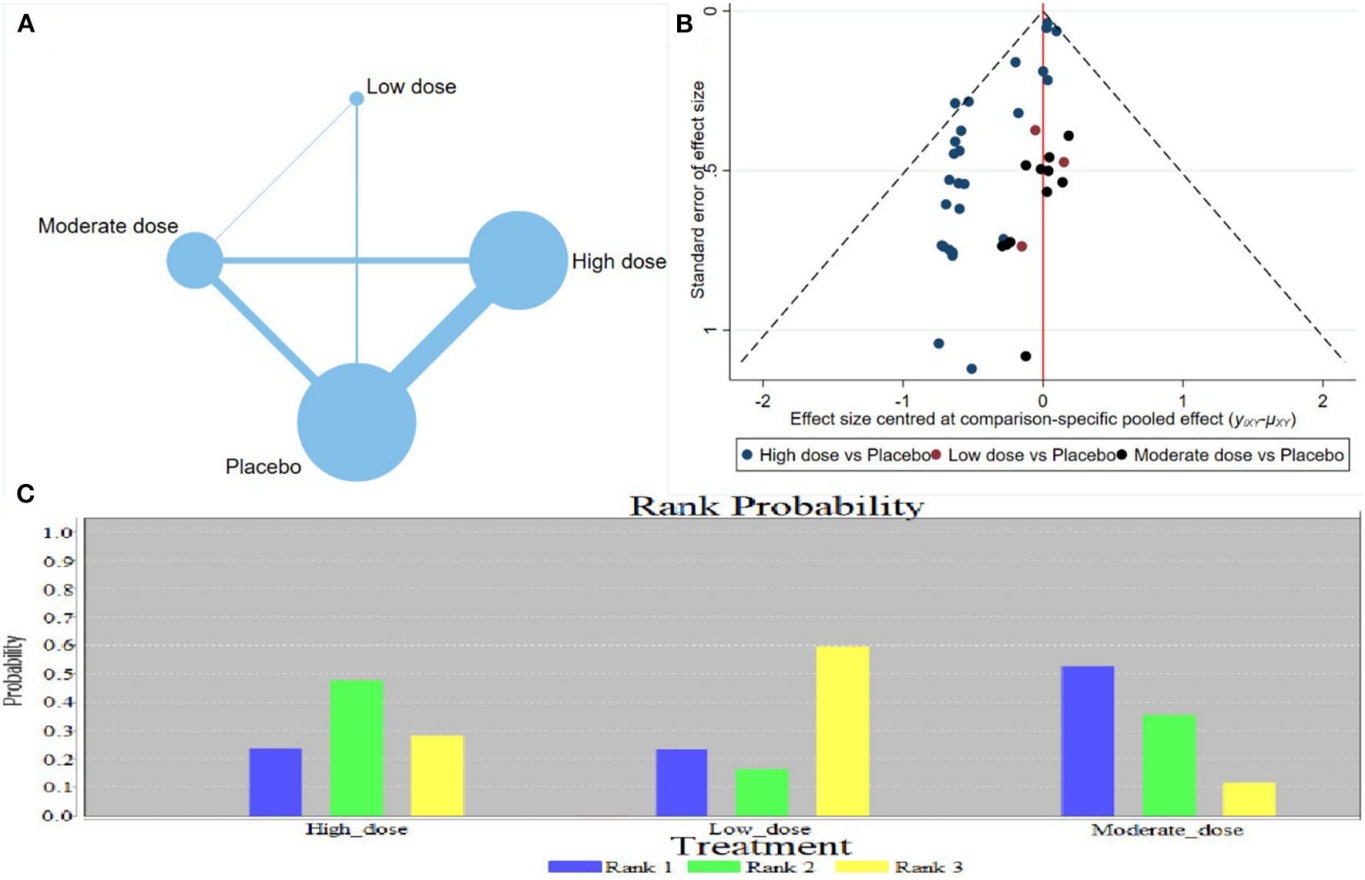
Results of network meta-analysis of gestational hypertension (low-risk population). **(A)** Evidence Network diagram; **(B)** the comparison-correction funnel plot; **(C)** the ranking results.

The network meta-analysis revealed that the different doses of calcium supplementation reduced the gestational hypertension incidence; however, no significant difference was observed in various doses (Table 2) [low-risk population: High dose vs. Medium dose = 0.89 (0.55, 1.43); high-risk population: High dose vs. Medium dose = 1.36 (0.07, 45.0)]. Evidence Network showed that a high dose of calcium supplementation showed a trend in preventing gestational hypertension (Figure 3A). The comparison–correction funnel plot was asymmetric, suggesting that the publication bias and small sample effect may exist (Figure 3B). The ranking results revealed that the three doses of calcium supplementation significantly reduced the incidence of gestational hypertension; however, the effect of medium dose may be the most effective (Figure 3C).

**Table 2:** Results of network meta-analysis of gestational hypertension.

**Low-population**	**High-risk population**				**High-population**
	**High _ dose**	1.36 (0.07, 45.0)➁	0.31 (0.01, 16.0)	0.13 (0.01, 1.20)	
	0.89 (0.55, 1.43)➀	**Medium _ dose**	0.23 (0.01, 4.61)	0.10 (0.01, 0.58)	
	1.18 (0.46, 2.96)	1.32 (0.52, 3.35)	**Low _ dose**	0.42 (0.01, 7.41)	
	0.31 (0.22, 0.42)	0.27 (0.11, 0.64)	0.35 (0.23, 0.54)	**Placebo**	
	**Low-risk population**				

*Interpretation of results: ➀ low-risk population: high dose vs. medium dose = 0.89 (0.55,1.43); ➁ high-risk population: high dose vs. medium dose = 1.36 (0.07, 45.0)*.

#### Gestational Hypertension (High Risk)

A total of 5 studies (25, 33, 52, 57, 65) reported the effect of calcium supplementation on preventing gestational hypertension.

The traditional meta-analysis showed that medium and high doses of calcium supplementation reduced the incidence of gestational hypertension [medium dose: *n* = 3 studies, RR = 0.16 (0.06, 0.44); high dose: *n* = 2 studies, RR = 0.34 (0.14, 0.84)] (Table 1). As only one study reported the effect of low-dose calcium supplementation in reducing gestational hypertension, the data could not be combined by the traditional meta-analysis (25).

**Figure 4:**
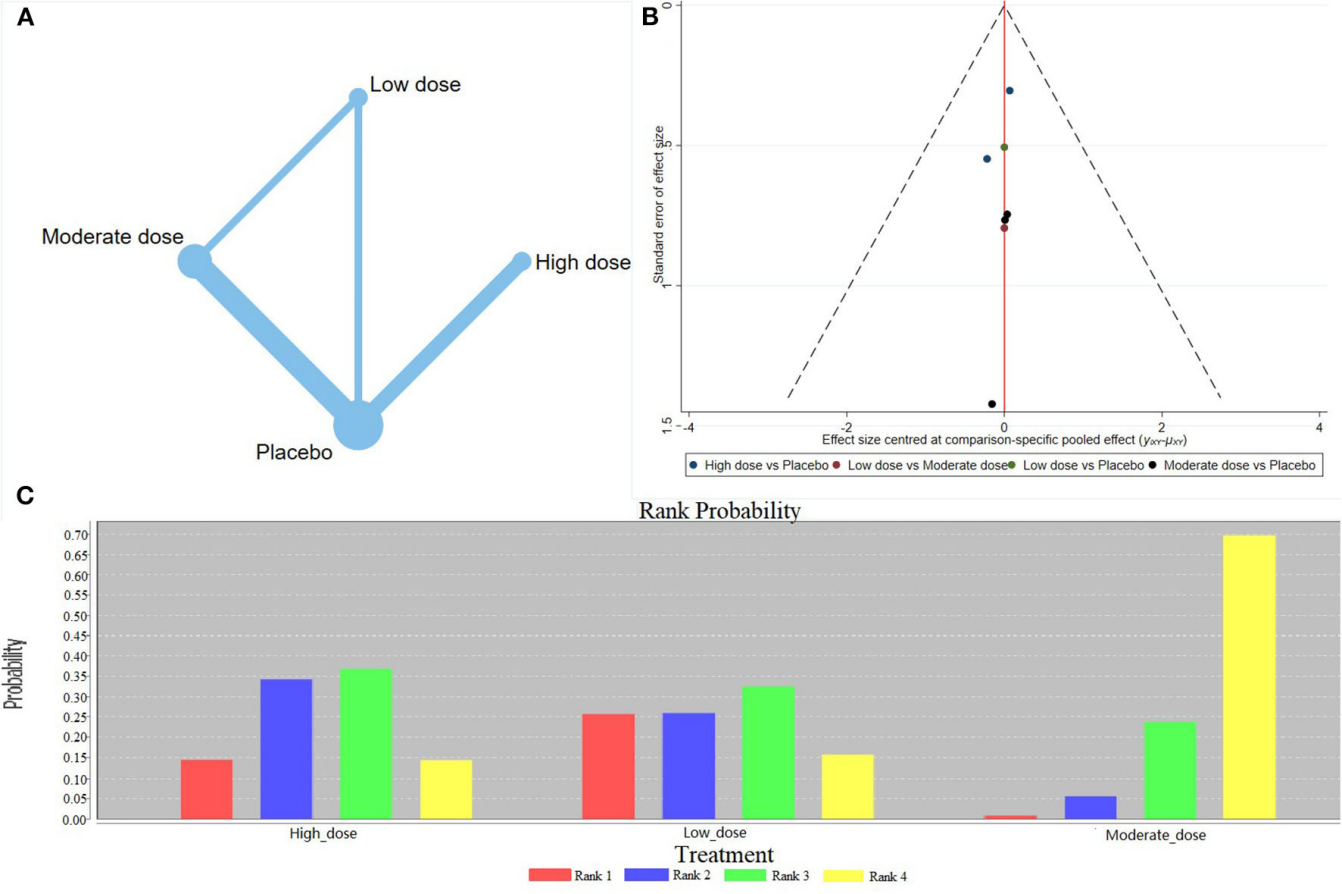
Results of network meta-analysis of gestational hypertension (high-risk population). **(A)** Evidence Network diagram; **(B)** the comparison-correction funnel plot; **(C)** the ranking results.

The network meta-analysis revealed that the medium doses of calcium supplementation reduced the incidence of gestational hypertension. Conversely, both low and high doses could not reduce the incidence of gestational hypertension. Of note, no significant difference was observed among different doses in reducing the incidence of gestational hypertension (Table 2). Evidence Network showed that a medium dose of calcium supplementation effectively prevented gestational hypertension (Figure 4A). The comparison–correction funnel plot was symmetrical, suggesting that publication bias was less likely (Figure 4B). However, the ranking results showed that a low dose of calcium supplementation was most effective in reducing gestational hypertension (Figure 4C).

#### Pre-Eclampsia (Low Risk)

A total of 20 studies (14–17, 40, 47, 49–51, 53, 55, 58–66) reported the effect of calcium supplementation on preventing the pre-eclampsia.

The traditional meta-analysis showed that different doses of calcium supplementation reduced the incidence of pre-eclampsia [low dose: *n* = 4 studies, RR = 0.49 (0.28, 0.85); medium dose: *n* = 3 studies, RR = 0.32 (0.15, 0.70); high dose: *n* = 13 studies, RR = 0.67 (0.52, 0.85)] (Table 3).

**Table 3:** Results of a traditional meta-analysis of pre-eclampsia.

**Pre-eclampsia**	**Risk level of disease**	**Number of studies**	**Model**	**RR**	***I*-squared**	***P***
Low dose vs. Placebo	Low	4	Random	0.49 [0.28,0.85]	70.8%	0.016
Medium dose vs. Placebo	Low	3	Fixed	0.32 [0.15,0.70]	0.0%	0.521
High dose vs. Placebo	Low	13	Random	0.67 [0.52,0.85]	64.4%	0.001
High dose vs. Placebo	High	3	Fixed	0.21 [0.09,0.50]	0.0%	0.578

**Figure 5:**
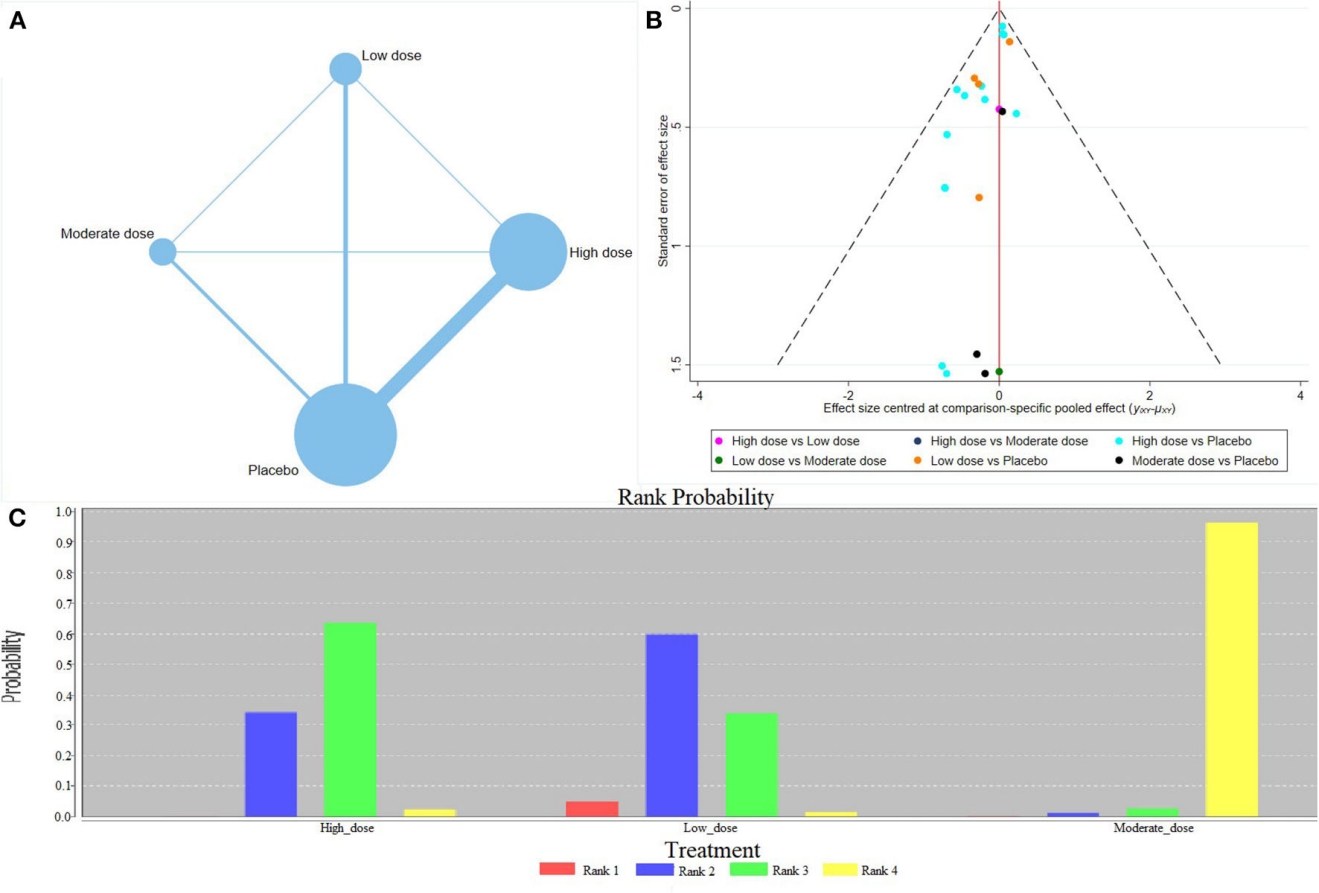
Results of network meta-analysis of pre-eclampsia (low-risk population). **(A)** Evidence Network diagram; **(B)** the comparison-correction funnel plot; **(C)** the ranking results.

The network meta-analysis showed that medium and high doses of calcium supplementation reduced the incidence of pre-eclampsia. Conversely, the low dose could not reduce the incidence of pre-eclampsia. The effect of medium and high doses of calcium supplements was significantly better than the low doses (Table 4) (The interpretation of results is similar to Table 2). Evidence Network revealed that a high dose of calcium supplementation was majorly effective in preventing pre-eclampsia (Figure 5A). The comparison–correction funnel plot was asymmetric, suggesting that publication bias and a small sample effect exist (Figure 5B). The ranking results showed that a low dose of calcium supplementation was most effective in reducing pre-eclampsia (Figure 5C). However, ranking results were less significant because they were significantly better than low at medium and higher doses.

**Table 4:** Results of network meta-analysis of pre-eclampsia (low-risk population).

**High _ dose**			
3.92 (0.93, 20.6)	**Medium _ dose**		
0.86 (0.37, 0.82)	0.22 (0.04, 0.95)	**Low _ dose**	
0.47 (0.27, 0.72)	0.12 (0.02, 0.45)	0.55 (0.26, 1.15)	**Placebo**

#### Pre-Eclampsia (High Risk)

A total of three studies (52, 54, 57) reported the effect of calcium supplementation on preventing pre-eclampsia. Pregnant women in these three studies were only given high doses of calcium supplementation. Therefore, we only obtained data from studies in which high-dose calcium supplementation was compared with placebo. Only a traditional meta-analysis could be carried out on the extracted data for this condition. The results showed that a high dose of calcium supplementation could reduce the incidence of pre-eclampsia [high dose: *n* = 3 studies, RR = 0.21 (0.09, 0.50)] (Table 3).

## Discussion

Compared with the previously published meta-analysis, our study compiled all the relevant literature of the eight Chinese and English databases. According to the inclusion and exclusion criteria formulated in advance, the literature was strictly screened. Finally, data of 48 comprehensive studies were included for the final merger analysis. Meanwhile, we grouped these populations into two risk levels: low risk and high risk. For subgroup analysis, the population was further grouped into three levels of a high, medium, and low, based on the calcium doses. Due to the lack of evidence for direct comparison between different calcium doses, we indirectly compared the effectiveness of different calcium doses in reducing gestational hypertension and pre-eclampsia by network meta-analysis to find evidence of the optimal calcium dose. Overall, our meta-analysis results were more stable and reliable.

### Overview of Evidence

For gestational hypertension, the results of the traditional meta-analysis were consistent with the network meta-analysis for the low-risk population, indicating that different doses of calcium supplementation reduced the incidence of gestational hypertension. In the low-risk groups, the low dose of calcium supplementation had met the calcium demand of pregnant women, and excessive calcium doses would inhibit iron absorption, thus affecting the overall development of the disease. Therefore, excessive doses of calcium supplementation did not show significant extended benefits (67, 68). The ranking results showed that a medium calcium dose had the best effect on preventing gestational hypertension. Therefore, medium calcium doses might be the best option for preventing gestational hypertension in a low-risk population. Our results were consistent with the meta-analysis of Hofmeyr et al., where they reported that calcium supplementation (≥1 g/day) reduced the risk of gestational hypertension (10). However, they did not distinguish the effects based on the doses of calcium supplementation. Besides, in their study, some participants were given other supplementation, such as linoleic acid and antioxidants, magnesium. Thus, it was difficult to isolate the actual therapeutic effect of calcium supplementation. On this basis, the calcium supplementation doses were further divided into different grades. However, we only included studies with calcium supplementation alone and placebo group. Our findings provided greater visibility on the optimal dose of calcium supplementation, which would be meaningful for guiding future clinical practice. In high-risk populations, this result of the traditional meta-analysis was not consistent with the network meta-analysis. The results of the traditional meta-analysis showed that both medium and high doses of calcium supplementation reduced the incidence of gestational hypertension. Whereas, network meta-analysis revealed that only a medium dose could reduce the incidence of gestational hypertension. Notably, neither high nor low doses of calcium supplementation could reduce the incidence of gestational hypertension. Meanwhile, contradictions in the results of the network meta-analysis also existed. The network meta-analysis revealed that only a medium dose of calcium supplementation reduced the incidence of gestational hypertension, however, with no significant difference in variable doses of calcium supplementation. Inconsistent results between the studies may be attributed to the differences in some baseline characteristics (including age, initiation of calcium supplementation, and high-risk factors). This inconsistency caused the substantial heterogeneity between included studies, which eventually led to the inconsistent results between the directly merged meta-analysis and the indirectly compared meta-analysis. In addition, only five studies reported the high-risk groups; thus, the small number of studies and sample size were prone to small sample bias, leading to differences in results. Therefore, based on our traditional and network meta-analysis results, the medium dose might be most effective for preventing gestational hypertension in high-risk groups.

For pre-eclampsia, the results of our traditional meta-analysis were consistent with the previous reports by Sun et al., where the different doses of calcium supplementation could reduce the incidence of pre-eclampsia in low-risk groups (20). Although Sun et al. divided the calcium supplementation doses (low dose: <0.6 g/day; medium dose: 0.6–1.2 g/day; and high dose: 1.2–2.0 g/day), the prophylactic effects between different calcium supplementation doses were not compared, which could not provide sufficient guidance for the future clinical practice. We performed a network meta-analysis to study the preventive effect of different calcium supplementation doses. In contrast to the results of traditional meta-analysis, the network meta-analysis results showed that only medium and high doses of calcium supplementation could reduce the incidence of pre-eclampsia. These results may be due to the inclusion of a significant number of pregnant teens, whose demand for calcium exceeds that of a pregnant adult because of continued maternal bone mineralization (17, 69). In addition, pre-eclampsia was a more serious HDP. Therefore, a low dose of calcium supplementation was ineffective in fulfilling the need for preventing pre-eclampsia.

In summary, both medium and high doses of calcium supplements prevented pre-eclampsia in low-risk populations; however, given the economic cost and drug side effects, a medium dose was the most effective in preventing pre-eclampsia (70, 71). On the other hand, only a high dose of calcium supplementation could prevent pre-eclampsia in high-risk groups. This finding was consistent with the results of Hofmeyr et al. where the high dose of calcium supplementation reduced the risk of pre-eclampsia, especially for high-risk pregnant women (10). However, their findings were susceptible to a small sample size and publication bias. In conclusion, there were few studies on calcium supplementation-mediated prevention of pre-eclampsia in high-risk groups. Therefore, large-sample and high-quality studies are required in the future.

### Quality of Evidence

Our study found that calcium supplementation for gestational hypertension and pre-eclampsia was not substantial based on a rigorous systematic review of current literature. Besides, the reliability of the results was reduced, which eventually reduced the authenticity of meta-analysis results. Probable reasons for this observation are discussed as follows:

1. **Heterogeneity of the included studies:** In high-risk groups, the baseline characteristics of maternal age (including 18–30 years old), initiation of calcium supplementation (including 14–30 weeks of pregnancy), and high-risk factors (including BMI ≥ 24, calcium deficiency symptoms, pre-eclampsia history, a positive roll-over test) were significantly different. These factors eventually caused more significant heterogeneity among the studies and reduced the reliability of meta-analysis results.
2. **Inadequate rigorous and scientific design of studies:** Among the 48 included studies, the random grouping method of 64.58% (31/48) of the studies was unclear, whereas 75% (36/48) of the studies did not report the implementation of covert grouping. This discrepancy led to the high likelihood of selective bias. Thus, a stricter grouping method should be considered for future research to reduce selection bias.
3. **Inclusion studies lack important quality control measures to reduce measurement and implementation bias:** A total of 62.5% (30/48) of the studies did not report blinding methods for patients, researchers, and outcome evaluators. For the determination of the outcome index, an effective scientific blinding method could avoid the influence of measurement bias on the measurement results, the qualification of the surveyors, the consistency of the surveyor's cognition of different outcome indicators, the accuracy, and scientific nature of the effectiveness criteria could affect the determination of the results in varying degrees. However, none of the 48 studies reported the qualifications of the surveyors or the standards and specific measurement processes used in the study. Therefore, future research should apply the blinding method for the experimental designs and report specific and comprehensive experimental details. This practice would improve the reproducibility and reliability of study results.
4. **Unbiased report of study conditions and data:** Although the included 48 studies reported all the predetermined results in their publications, only 18.75% of the studies reported the research protocols. Due to this discrepancy, it was impractical to finally judge whether to report all their results as per the plan and without bias. Selective reporting of the research results could lead to publication bias, which eventually affects the reliability of systematic review conclusions and even prompts an opposite conclusion. In addition, although most studies have reported calcium supplementation, the specific components of calcium supplements (including calcium carbonate, calcium chelate, and calcium tablets) were not reported. The variation in the formulation may result in a deviation between the reported and the real dose of calcium supplementation for pregnant women, thus affecting the reliability of the conclusions of our study (72).

In conclusion, despite the comprehensive analysis of basic information, risk of bias, outcome measures, and other aspects, the reliability of meta-analysis results was reduced due to the limitations of current research in study design, result measurement, statistics, and evidence quality. However, our study has confirmed that medium dose was the most effective dose of calcium supplement to prevent gestational hypertension (low-risk and high-risk populations) and pre-eclampsia (low-risk population). Unfortunately, only a few studies are reported on preventing gestational hypertension and pre-eclampsia for high-risk groups. Therefore, more high-quality clinical studies are required to further explore the specific role of calcium. Moreover, future research should be diligently carried out in study design, implementation, measurement, and evaluation of results and research protocols, to improve the quality of studies.

### Advantages and Limitations of This Study

Advantages of this study: (1) The subgroup analysis of the included population was carried out for the different risk groups and calcium supplementation doses, which improved the applicability of the meta-analysis results. (2) An indirect comparison of the effects of different calcium doses yielded evidence for optimal calcium doses. (3) As more studies were included, the conclusion was more comprehensive and reliable.

Limitations of this study: (1) Most studies did not report daily dietary calcium intake in pregnant women. Thus, our study could not consider the effect of dietary calcium intake on calcium supplementation during pregnancy. Moreover, the people with adequate dietary calcium intake might have a smaller response to the effect of calcium supplementation (17, 69). (2) The number of studies on high-risk gestational hypertension (five studies) and pre-eclampsia (three studies) were small. Therefore, the meta-analysis results based on studies with a small sample size need to be treated with caution. (3) Only Chinese and English databases were queried, leading to language bias. (4) Gray literature and conference abstracts were not included in the analysis, leading to publication bias. (5) The registration of the systematic review protocol is an important measure to improve transparency, avoid bias, and repeat the publication of results. Although we reported the specific research process and results, we could not register our protocol in advance in any database.

## Conclusion

All doses of calcium could reduce the incidence of gestational hypertension in a low-risk population, whereas medium dose could reduce the incidence of gestational hypertension in high-risk groups. Moreover, in the comprehensive analysis of the included studies, the medium dose was most effective (low-risk and high-risk populations). Both medium and high doses of calcium could reduce the incidence of pre-eclampsia in low-risk groups. However, a medium dose was found to have maximum effect. The bias was caused by the limitations of included studies regarding outcome measurement and reporting, whereas the fewer data on some indicators reduced the authenticity and reliability. Therefore, future trials need to design research programs more scientifically and rationally by including large cohorts. This intervention would reduce the risk of bias, improve the quality of evidence, and further evaluate the efficacy of calcium supplementation.

## Data Availability Statement

The original contributions presented in the study are included in the article/Supplementary Material, further inquiries can be directed to the corresponding author/s.

## Author Contributions

RH contributed to the design, guidance, and modification of the project. DC and HW completed the implementation and writing of the paper. XX, LZ, AY, and SL completed the collection and collation of the data. All authors contributed to the article and approved the submitted version.

## Conflict of Interest

The authors declare that the research was conducted in the absence of any commercial or financial relationships that could be construed as a potential conflict of interest.

## Publisher's Note

All claims expressed in this article are solely those of the authors and do not necessarily represent those of their affiliated organizations, or those of the publisher, the editors and the reviewers. Any product that may be evaluated in this article, or claim that may be made by its manufacturer, is not guaranteed or endorsed by the publisher.
