# Supplementary material for: Different Doses of Calcium Supplementation to Prevent Gestational Hypertension and Pre-Eclampsia: A Systematic Review and Network Meta-Analysis
Source: Front Nutr. 2022 Jan 17;8:795667. doi: 10.3389/fnut.2021.795667 (PMC8801486; doi:10.3389/fnut.2021.795667)
Supplement: Supplementary file 1 [file Table_1.docx]

**Basic information for inclusion in the study**

| **Author (year)** | **Country** | **Type of study** | **Age(year)** | **Start time of calcium supplement (week)** | **Risk of disease** | **Sample** | | **Dietary calcium intake** | **Dosage/d** | | **Number of loss to follow-up and the reasons** |
| --- | --- | --- | --- | --- | --- | --- | --- | --- | --- | --- | --- |
|  |  |  |  |  |  | **Experimental** | **Control** |  | **Experimental** | **Control** |  |
| Jing Zhang 2008 | China | RCT | 27.55±4.35(20-39) | 20 | low | 60 | 60 | / | 0.5g calcium | Blank | 0 |
| Guiyuan Li 2004 | China | RCT | 22-35 | 20-28 | low | 120 | 120 |  | 500mg calcium carbonate | Blank | 0 |
| Lihong Zhou 2004 | China | RCT | <35 | 20 | low | 50 | 50 | / | 2g calcium amino acid chelate | Blank | 0 |
| Yuan Zhang 2004 | China | RCT | 21-35 | 20 | low | 23 | 27 | / | 1g calcium amino acid chelate | Blank | 0 |
| Yuehui Wu 2003 | China | RCT | 21-36 | 20-32 | low | 100 | 100 | / | 2g calcium amino acid chelate | Blank | 0 |
| Yanyuan Wang 2000 | China | RCT | 22-37 | 26-28 | low | 165 | 82 | / | 3g calcium | Blank | 0 |
| Hui Wang 2008 | China | RCT | 21-32 | 20-28 | low | 50 | 50 | / | 2g calcium amino acid chelate | Blank | 0 |
| Hongmei Shi 2006 | China | RCT | 22-34 | 20 | low | 50 | 50 | / | 2g calcium amino acid chelate | Blank | 0 |
| Qiue Niu 2007 | China | RCT | 21-35 | 20-24 | low | 60 | 60 | / | 2g calcium amino acid chelate | Blank | 0 |
| Hongping Ma 2006 | China | RCT | 24.5±0.4(23-30) | 24±0.5 | low | 201 | 201 | / | 2g calcium amino acid chelate | Blank | 0 |
| Li Liu 2009 | China | RCT | 26.5±0.4(21-30) | 25±0.5(22-28) | low | 402 | 402 | / | 2g calcium amino acid chelate | Blank | 0 |
| Xuelan Li 2000 | China | RCT | / | 20-24 | High (BMI≥24) | 29 | 30 | 505.32±189.36mg | 1200mg/600mg calcium | Blank | 0 |
| Shaoxiong Li 2010 | China | RCT | 22-36 | 26-34 | low | 50 | 50 | / | 1.2g calcium amino acid chelate | Blank | 0 |
| Yingyu Lei 2012 | China | RCT | 26.5±3.7/26.1±2.9 | 17-24 | low | 100 | 100 | / | 1g calcium amino acid chelate | Blank | 0 |
| Xiulian Lan 2005 | China | RCT | 20-35 | 16-28 | low | 100 | 100 | / | 2g calcium amino acid chelate | Blank | 0 |
| Weiyan He 2008 | China | RCT | 25.5±0.4(24-30) | 24±0.5(20-26) | low | 158 | 129 | / | 2g calcium amino acid chelate | Blank | 0 |
| Qiaohong Gong 2005 | China | RCT | 20-30 | 14-30 | High (symptoms of calcium deficiency) | 80 | 80 | / | 1g calcium amino acid chelate | Blank | 0 |
| Jing Xiang 2004 | China | RCT | 21-35 | 20-33 | low | 50 | 50 | / | 2g; 1g calcium amino acid chelate | Blank | 0 |
| Gu li 2009 | China | RCT | 20-34 | 24-28 | low | 60 | 60 | / | 2g; 1g calcium amino acid chelate | Blank | 0 |
| Yunqiao Song 2007 | China | RCT | 20-35 | 20 | low | 80 | 80 | / | 2g; 1g calcium amino acid chelate | Blank | 0 |
| Hongjun Sun 1999 | China | RCT | / | / | low | 86 | 86 | / | 2g; 1g calcium | Blank | 0 |
| Xiaomei Liu2009 | China | RCT | 20-35 | 20-34 | low | 54 | 52 |  | 2g; 1g calcium | Blank | 0 |
| Fang Yang 2008 | China | RCT | 26±2.6/25±2.4 | 20-24 | low | 30 | 30 | 615.32±189.36 | 1.2g; 0.6g calcium | Blank | 0 |
| Ming Lin 2015 | China | RCT | 21-35 | 20-24 | low | 80 | 80 | / | 2g; 1g calcium amino acid chelate | Blank | 10 people lost to follow up for personal reasons |
| Taherian 2002 | Iran | RCT | 21.9±0.28(21.2±0.19) | 20 | low | 330 | 330 | / | 500 mgcalcium carbonate | Blank | 0 |
| Hofmeyr 2019 | South Africa | RCT | 29.4±5.4/29.2±5.1 | 20 | high | 298 | 283 | 1500mg | 500mg calcium carbonate | Placebo | 2 |
| Belizan 1991 | Argentina | RCT | 23.7±5.5/23.7±5.7 | 20 | low | 593 | 601 | / | 2gcalcium carbonate | Placebo | 27 women were lost to follow-up after randomization. 98 women were lost because of a change of hospital, physician, or residence |
| Bassaw 1998 | Trinidad | RCT | / | / | High (preeclampsia history) | 81 | 250 | / | 1.2g elemental calcium | Placebo | 0 |
| Khan 2013 | India | RCT | 18-30 | 20 | low | 127 | 145 | / | 2g elemental calcium | 0.5g calcium | 4/6 |
| Azar 2015 | India | RCT | 36.9±1.9/37.4±2.5 | 18-20 | low | 45 | 44 | / | 1g elemental calcium | Placebo | 5/4 |
| Cong 1995 | China | RCT | / | 20 | low | 212 | 106 | / | 2g; 1g; 0.24g; 0.12g calcium | Placebo | 0 |
| Crowther 1999 | Australia | RCT | 25.1±5.3/24.3±5.4 | 24 | low | 227 | 229 | / | 1.8g calcium | Placebo | Some women in each treatment group decided to stop taking the trial medication during the antenatal period, 70 (3 1 %) in the calcium group and 54 (24%) in the placebo group. The most frequent reason given was that the tablets were too large (26 (41%) calcium; 20 (39%) placebo). |
| Hiller 2007 | Australia | RCT | 25.8±5.4 | 20 | low | 105 | 104 | / | 1.8g calcium | Placebo | 0 |
| Hofmeyr 2008 | South Africa | RCT | 22±3.7/22.1±3.7 | 20 | low | 346 | 362 | / | 1.5g calcium carbonate | Placebo | 0 |
| Kumar 2009 | India | RCT | 21.83±2.51/21.91±2.47 | 12-25 | low | 273 | 251 | 85.71-910.71 | 2g calcium carbonate | Placebo | 0 |
| Levine 1997 | America | RCT | 21±4 | 13-21 | low | 2295 | 2294 | / | 2g calcium carbonate | Placebo | 253 women were lost to follow-up |
| López 1989 | Ecuador | RCT | 18.2±2.2/18.7±2.7 | 23 | low | 55 | 51 | / | 2g elemental calcium | Placebo | 14 people lack the final outcome measurement data |
| López 1990 | Ecuador | RCT | 19.4±1.8 | 28-32 | High (a positive roll-over test) | 22 | 34 | / | 2g elemental calcium | Placebo | 0 |
| López 1997 | Ecuador | RCT | 16.1±0.6/15.9±0.7 | 20 | low | 125 | 135 | 628±302/605±421 | 2g calcium carbonate | Placebo | 0 |
| Nenad 2011 | Serbia | RCT | / | 14-23 | low | 4590 | 4588 | / | 2g calcium | Placebo | 0 |
| Niromanesh 2001 | Iran | RCT | 23.8±4.7/22.5±4.9 | 28-32 | High (having positive results on the rollover test and having at least one risk factor for pre-eclampsia) | 15 | 15 | / | 2g calcium | Placebo | 0 |
| Villar 2006 | Argentina | RCT | 22.6±4.4/22.7±4.4 | 20 | low | 4157 | 4168 | 600mg | 1.5g calcium | Placebo | 13 women were excluded before intervention, 298 were lost to follow-up without delivery information, but the available data were included for other outcomes. |
| Purwar 1996 | India | RCT | 21.78±2.31/22.08±2.87 | 20 | low | 97 | 93 | 336±156/352±142 | 2g calcium | Placebo | 0 |
| Villar 1990 | America | RCT | 16.2±0.8/16.3±0.9 | 20 | low | 95 | 95 | 1201±521/1248±610 | 2g calcium | Placebo | 12 |
| Sanchez 1994 | America | RCT | 18.0±3.6/18.7±3.2 | 24-28 | high | 29 | 34 | 630.2±217.4/666.1±225.6 | 2g calcium carbonate | Placebo | 11 people who fail to comply with the agreement will refuse to withdraw from the follow-up |
| Wanchu 2001 | India | RCT | 37.2 | 20 | low | 50 | 50 | / | 2g calcium | Placebo | 0 |
| Villar 1987 | Argentina | RCT | 21.0±3.1/21.2±3.6 | 26 | low | 25 | 27 | / | 1.5g calcium | Placebo | 0 |
| Almirante 1998 | Philippines | RCT | / | 16-20 | high | 210 | 212 | / | 0.5g calcium | Placebo | 0 |
