# Supplementary material for: Different Doses of Calcium Supplementation to Prevent Gestational Hypertension and Pre-Eclampsia: A Systematic Review and Network Meta-Analysis
Source: Front Nutr. 2022 Jan 17;8:795667. doi: 10.3389/fnut.2021.795667 (PMC8801486; doi:10.3389/fnut.2021.795667)
Supplement: Supplementary file 2 [file Data_Sheet_1.docx]

**Supplemental data:**

**Chinese and English search strategies**

Comments:

In order for non - Chinese readers to understand the Chinese search strategy of this article, we translated the Chinese search terms in the search formula.

**1：CNKI：1281**

(SU=妊娠期高血压疾病 OR SU=妊高症 OR SU=妊娠期高血压 OR SU=妊娠高血压 OR SU=子痫 OR SU=子痫前期 OR SU=妊娠合并慢性高血压 OR SU=慢性高血压并发子痫前期 OR SU=HDCP) AND (SU=钙 OR SU=Ca)

(SU= Hypertensive disorders of pregnancy OR SU= pregnancy hypertension syndrome OR SU= gestational hypertension OR SU= hypertension of pregnancy OR SU= eclampsia OR SU= preeclampsia OR SU= pregnancy with chronic hypertension OR SU= chronic hypertension complicated with preeclampsia OR SU=HDCP) AND (SU=calcium OR SU=Ca)

**2.Wanfang 1957**

#1: 妊娠期高血压疾病 OR 妊高症 OR 妊娠期高血压 OR 妊娠高血压 OR 子痫 OR 子痫前期 OR 妊娠合并慢性高血压 OR 慢性高血压并发子痫前期

#2: 钙 OR Ca

#3: #1 AND #2

#1: Hypertensive disorders of pregnancy OR pregnancy hypertension syndrome OR gestational hypertension OR hypertension of pregnancy OR eclampsia OR preeclampsia OR pregnancy with chronic hypertension OR chronic hypertension complicated with preeclampsia

#2: calcium OR Ca

#3: #1 AND #2

**3. VIP 455**

#1: 妊娠期高血压疾病 OR 妊高症 OR 妊娠期高血压 OR 妊娠高血压 OR 子痫 OR 子痫前期 OR 妊娠合并慢性高血压 OR 慢性高血压并发子痫前期

#2: 钙 OR Ca

#3: #1 AND #2

#1: Hypertensive disorders of pregnancy OR pregnancy hypertension syndrome OR gestational hypertension OR hypertension of pregnancy OR eclampsia OR preeclampsia OR pregnancy with chronic hypertension OR chronic hypertension complicated with preeclampsia

#2: calcium OR Ca

#3: #1 AND #2

**4.CBM 1272**

#1: "子痫"[不加权:扩展] 2691

#2: "妊娠期高血压疾病"[常用字段:智能] OR "妊高症"[常用字段:智能] OR "妊娠期高血压"[常用字段:智能] OR "妊娠高血压"[常用字段:智能] OR "子痫"[常用字段:智能] OR "子痫前期"[常用字段:智能] OR "妊娠合并慢性高血压"[常用字段:智能] OR "慢性高血压并发子痫前期"[常用字段:智能] 38556

#3: (#2) OR (#1) 38556

#4: "钙"[不加权:扩展] 63625

#5: 钙"[常用字段:智能] OR "Ca"[常用字段:智能]243009

#6: (#5) OR (#4) 286936

#7: #3 AND #6 1272

#1: " eclampsia "[unweighted, extended]2691

#2: " Hypertensive disorders of pregnancy "[common field: smart] OR " pregnancy hypertension syndrome "[common field: smart] OR " gestational hypertension "[common field: smart] OR " hypertension of pregnancy "[common field: smart] OR " eclampsia "[common field: smart] OR " preeclampsia "[common field: smart] OR " pregnancy with chronic hypertension "[common field: smart] OR " chronic hypertension complicated with preeclampsia "[common field: smart] 38556

#3: (#2) OR (#1) 38556

#4: " calcium "[unweighted, extended]63625

#5: calcium "[common field: smart] OR "Ca"[common field: smart]243009

#6: (#5) OR (#4) 286936

#7: #3 AND #6 1272

**5.Pubmed 1373**

#1: (("Eclampsia"[Mesh]) OR "Pre-Eclampsia"[Mesh]) OR "Hypertension, Pregnancy-Induced"[Mesh] 38369

#2: pregnancy-induced hypertension[Title/Abstract] OR pregnancy induced hypertension[Title/Abstract] OR gestational hypertension[Title/Abstract] OR chronic hypertension with superimposed preeclampsia[Title/Abstract] OR eclampsia[Title/Abstract] OR preeclampsia[Title/Abstract] OR hypertensive disorder complicating pregnancy[Title/Abstract] OR pregnancy hypertension[Title/Abstract] OR HDCP[Title/Abstract] OR hypertension of pregnancy[Title/Abstract] OR (pregnancy[Title/Abstract] AND chronic hypertension[Title/Abstract]) 41440

#3: #1 OR #2 54980

#4: "Calcium"[Mesh] 273253

#5: calcium [Title/Abstract] OR Ca [Title/Abstract] 613014

#6: #4 OR #5 697877

#7: #3 AND #6 1373

**6.Embase 3501**

#1: 'pregnancy-induced hypertension'/exp OR 'pregnancy-induced hypertension' 22617

#2: 'gestational hypertension'/exp OR 'gestational hypertension' 21460

#3: 'eclampsia'/exp OR 'eclampsia' 27484

#4: 'preeclampsia'/exp OR 'preeclampsia' 69623

#5: 'pregnancy hypertension'/exp OR 'pregnancy hypertension' 21904

#6: 'pregnancy-induced hypertension':ab,ti OR 'pregnancy induced hypertension':ab,ti OR 'gestational hypertension':ab,ti OR 'chronic hypertension with superimposed preeclampsia':ab,ti OR eclampsia:ab,ti OR preeclampsia:ab,ti OR 'hypertensive disorder complicating pregnancy':ab,ti OR 'pregnancy hypertension':ab,ti OR hdcp:ab,ti OR 'hypertension of pregnancy':ab,ti OR 'pregnancy with chronic hypertension':ab,ti 60263

#7: #1 OR #2 OR #3 OR #4 OR #5 OR #6 87821

#8: 'calcium'/exp OR 'calcium' 869833

#9: calcium:ab,ti OR ca:ab,ti 824969

#10: #8 OR #9 1079385

#11: #9 AND #10 3501

**7.WOS 1754**

TS=(pregnancy-induced hypertension OR pregnancy induced hypertension OR gestational hypertension OR chronic hypertension with superimposed preeclampsia OR eclampsia OR preeclampsia OR hypertensive disorder complicating pregnancy OR pregnancy hypertension OR HDCP OR hypertension of pregnancy OR pregnancy with chronic hypertension) AND TS=( calcium OR Ca)

**8. Cochrane 388**

#1: MeSH descriptor: [Hypertension, Pregnancy-Induced] explode all trees 1188

#2: MeSH descriptor: [Eclampsia] explode all trees 200

#3: MeSH descriptor: [Pre-Eclampsia] explode all trees 1010

#4: (pregnancy-induced hypertension OR pregnancy induced hypertension OR gestational hypertension OR chronic hypertension with superimposed preeclampsia OR eclampsia OR preeclampsia OR hypertensive disorder complicating pregnancy OR pregnancy hypertension OR HDCP OR hypertension of pregnancy OR pregnancy with chronic hypertension):ti,ab,kw 6216

#5: #1 or #2 or #3 or #4 6237

#6: MeSH descriptor: [Calcium] explode all trees 3510

#7: (calcium OR Ca):ti,ab,kw 40976

#8: #6 or #7 40976

#9: #5 and #8 388
